# A mechanism of surface hardness enhancement for H^+^ irradiated polycarbonate

**DOI:** 10.1039/d0ra05073c

**Published:** 2020-08-04

**Authors:** Sunmog Yeo, Won-Je Cho, Dong-Seok Kim, Chan Young Lee, Yong Seok Hwang, Jae Kwon Suk, Chorong Kim, Jun Mok Ha

**Affiliations:** Korea Multi-purpose Accelerator Complex, Korea Atomic Energy Research Institute 38180 Republic of Korea sunmog@kaeri.re.kr

## Abstract

H^+^ irradiation increases the surface hardness of polycarbonate. Nano indentation measurement shows that the hardness increases up to 3.7 GPa at the dose of 5 × 10^16^ # cm^−2^ and at the irradiation energy of 150 keV. In addition, the hardness increases with the dose and the energy of H^+^ irradiation. In accordance with the nano indentation measurement, the Fourier-transform infrared spectroscopy (FTIR) depends on the dose and energy of H^+^ irradiation. The peak at ∼1500 cm^−1^ for the aromatic ring and the peak at ∼1770 cm^−1^ for the C=O stretch decrease with increasing dose and energy, while the increase of the dose and energy develops a new C=O stretch vibration at ∼1700 cm^−1^ and forms aromatic hydrocarbons at ∼1600 cm^−1^. X-ray diffraction experiments are also consistent with the nano indentation measurement and FTIR spectra. Based on the experiments, we discuss a possible mechanism of the surface hardness enhancements by ion beam irradiation.

## Abstract 2

H^+^ irradiation increases the surface hardness of polycarbonate.

## Graphical abstract

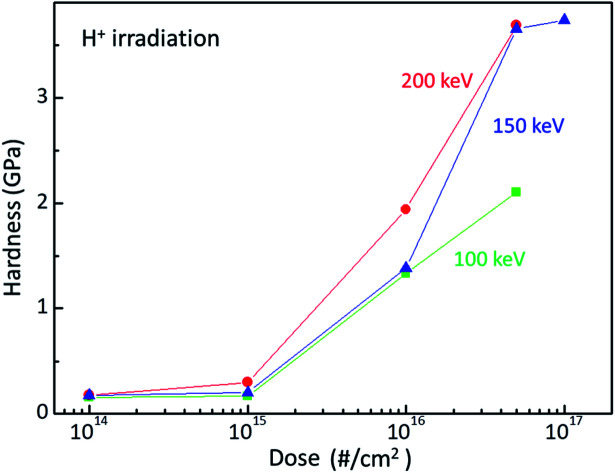

## Introduction

Polymers are of great research interest due to their versatile applications in areas such as vehicle light-weighting, fabrication of 3D micro patterned surfaces, optical sensors, and integrated optics for communications.^1–3^ Surface modification is an important aspect in polymer application and modification creates additional polymer properties. There are several ways to modify polymer surfaces, for example, *via* ion beam irradiation, electron beam irradiation, and coating. Especially, ion beam irradiation usually enhances the mechanical properties of polymers; it increases surface hardness while lowering the wear coefficients.^4–7^ One mechanism in which ion irradiation makes polymer surfaces hard is known as crosslinking,^8–12^ while nano-crystalline alloy is governed by different mechanism.^13^ A crosslink is a connecting bond between two polymer chains where the bond usually forms a covalent bond or ionic bond.^14^ Thus, when crosslinking occurs, polymer surface hardness tends to increase. However, to our best knowledge, there are few systematic studies related to the mechanism of surface hardness enhancement.

On the other hands, polycarbonate (PC) has high heat resistance, optical clarity, dimension stability, and outstanding electrical resistance. Therefore, PC is known to be a versatile engineering polymer which is used in mechanical components, electric appliances, automotive industry, and so forth. However, PC is intrinsically soft and easily scratched, and thus, surface treatments are required before applying to the field which requires high surface hardness with inherent PC properties such as in vehicle light-weighting and optical sensors.^15^ There are varied surface treatments for PC. These include coating, ultra-violet irradiation and ion beam irradiation. Among these, ion beam irradiation has certain advantages; it is environmentally friendly and improves surface hardness and wear resistance. There have been several papers on the surface hardness enhancement of PC through ion beam irradiation.^4–6^ However, the enhancement mechanism of the surface hardness remains unclear. Some of the research simply alludes to crosslinking or the formation of carboxyl groups. In order to better understand the mechanism, more systematic studies are required. In this paper, the surface hardness and Fourier-transform infrared spectroscopy (FTIR) spectra for ion beam irradiated PC are examined with increasing dose and irradiation energy. Moreover, we explain the mechanism of the surface hardness enhancement by comparing FTIR spectra for H^+^ irradiated PC.

## Experiment

### Ion beam irradiation

The samples used in the experiments were commercial PC. H^+^ ions were extracted from a duoPIGatron ion source and accelerated up to 200 keV through an electrostatic acceleration tube. By using a mass separation magnet, H^+^ ions were separated from other ions such as H_2_^+^ and H_3_^+^. The working pressure of the target chamber was below 5 × 10^−6^ torr. Faraday cups were used for measuring the dose of H^+^ ion. In order to certify the uniformity of H^+^ dose on the samples, we used two beam scanners; one scanner controlled up and down irradiation and the other scanner controlled left and right irradiation. Since the glass temperature of PC is about 147 °C, ion beam irradiation was perform at low current density (less than 1 μA cm^−2^). In addition, the sample stage was equipped with water cooling system so that the sample temperature did not exceed 60 °C during ion beam irradiation.

### Nano indentation test

Surface hardness was measured by an Anton Paar nano-indentation tester (model: CPX-UNHT). Measurements were performed more than five times based on the modulus mode with a Berkovich tip and the data shown in this paper are an averaged value. In addition, in order to check the effect of the damaged layer by ion beam irradiation, the sinus mode was used, where the minimum load and the maximum load were 0.05 mN and 20 mN, respectively. The sinus frequency and the maximum sinus amplitude were 5 Hz and 1 mN, respectively.

### FTIR spectra

The FTIR spectra of H^+^ irradiated PC were obtained by using an Agilent spectrometer (model: CARY 660) with an attenuated total reflectance device. Since the samples were thick, the spectra were acquired by using a reflection mode at a spectral resolution of 0.5 cm^−1^ in the wavenumber range from 4000 cm^−1^ to 650 cm^−1^. Note that the signal to noise ratio was checked with a vacuum-pressed KBr disc. In addition, in order to compare additional peaks after ion beam irradiation, the base lines of spectra were corrected using the Agilent FTIR spectrometer software.

### X-ray diffraction

A PANalytical (Empyrean) X-ray diffractometer with Cu Kα radiation was used to examine the PC structure. The wavelength of 1.5406 Å was generated from an X-ray radiation source of Cu Kα with the input power of 40 kV and 30 mA. A *θ*–2*θ* scan was performed in the step mode wherein the data acquisition time was 30 s at every data point.

## Results and discussion

**Fig. 1:**
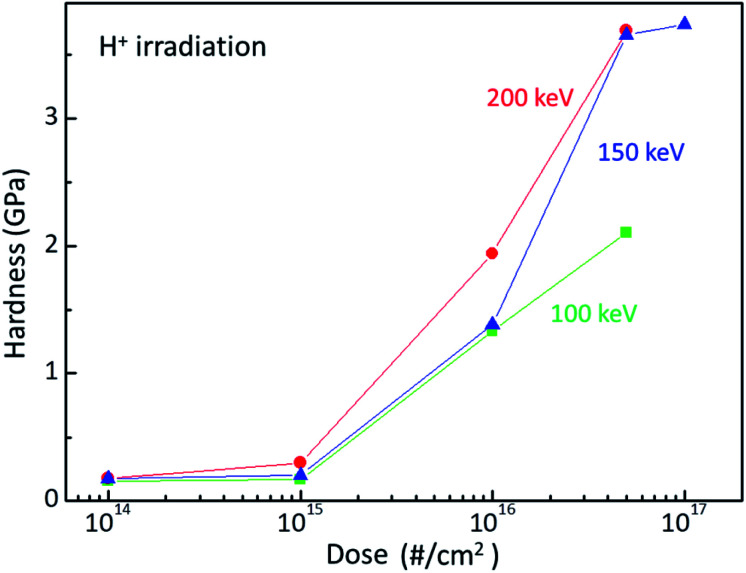
Surface hardness of H^+^ irradiated PC at the energy of 200 keV (red circles), 150 keV (blue triangles), and 100 keV (green squares).

Ion beam irradiation can increase the surface hardness of polymer at a certain dose and energy. Fig. 1 plots the surface hardness as a function of dose for H^+^ irradiated PC. The red circles, blue triangles, and green squares stand for the surface hardness values of H^+^ irradiated PC at the energies of 200 keV, 150 keV, and 100 keV, respectively. As expected, the surface hardness increases with increasing H^+^ dose. Up to the dose of 10^15^ # cm^−2^, the surface hardness shows a slight increase while the surface hardness increases rapidly at the dose of 10^16^ # cm^−2^ and 5 × 10^16^ # cm^−2^ for all the irradiation energies. Further irradiation such as 10^17^ # cm^−2^ at 150 keV, however, the surface hardness increases slightly. In fact, too much irradiation can make the polymer brittle. Thus, there is an effective dose range for surface hardness enhancement, which is from ∼5 × 10^15^ # cm^−2^ to ∼5 × 10^16^ # cm^−2^. Note that the hardness at the dose 5 × 10^16^ # cm^−2^ is about 3.7 GPa. In addition, one can notice the energy dependence at the same dose; surface hardness increases with increasing irradiation energy.

**Fig. 2:**
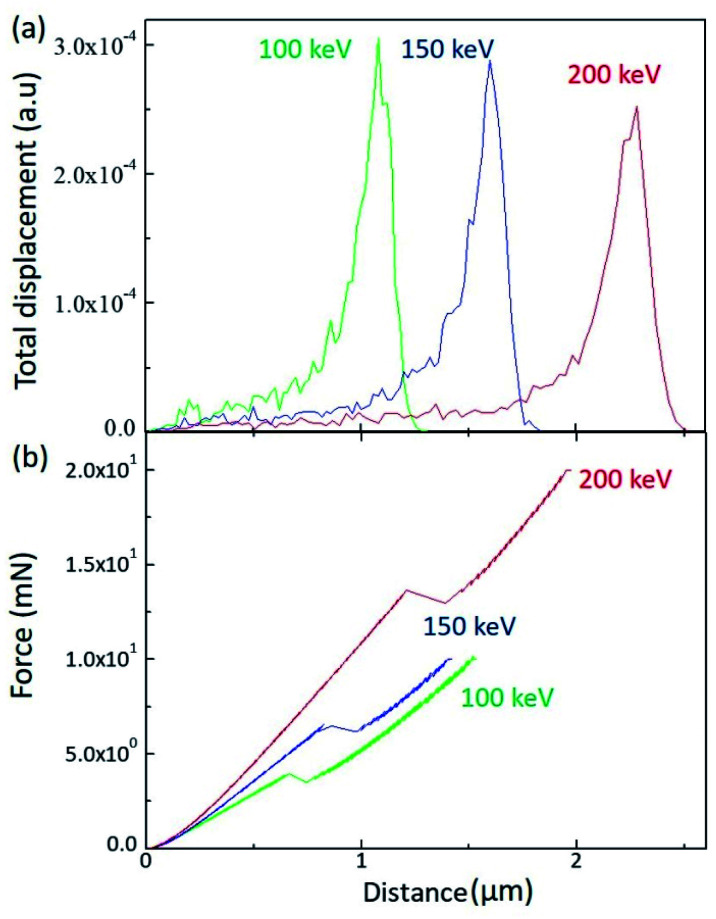
(a) SRIM calculation results for the different irradiation energy and (b) load as a function of depth by sinus mode indentation test.

In order to scrutinize the energy dependence, a SRIM (Stopping and Range of Ion in Matter) calculation was performed for the H^+^ irradiated PC, as shown in Fig. 2(a). When energetically charged particles pass through matter, they lose their energy by colliding with electrons and nuclei in the matter. Before they come to stop, they collide more and more frequently with nuclei and form a damaged layer at a certain depth range. Fig. 2(a) shows the depth range of the damaged layers for 100 keV (green line), 150 keV (blue line), and 200 keV (red line). As expected, the higher energy generates the deeper damaged layer in matter.

The damaged layer can be detected by a nano-indentation test by using a sinus mode. Fig. 2(b) displays the indentation load *versus* indentation depth for the different irradiation energy. Of note is that, when the indentation depth increases, there is a kink of the load at a certain depth, which is indicative of the existence of the damaged layer by ion irradiation. In addition, the depth of the load kink increases with increasing irradiation energy, which is consistent with the SRIM calculation. However, the kink depth is less than the SRIM estimation. Since the nano-indentation measurement is affected by the below layer, the kink depth proceeds to the damaged layer. As the SRIM calculation shows that the full width at half maximum increases with increasing irradiation energy, the kink depth width also increases with increasing irradiation energy. It is notable that the kink depth widths for 100 keV, 150 keV, and 200 keV are 0.08 μm, 0.14 μm, and 0.19 μm, respectively. In addition, the load slope increases with increasing irradiation energy, meaning that the PC surface hardness increases with increasing irradiation energy. This analysis is consistent with the surface hardness shown in Fig. 1.

**Fig. 3:**
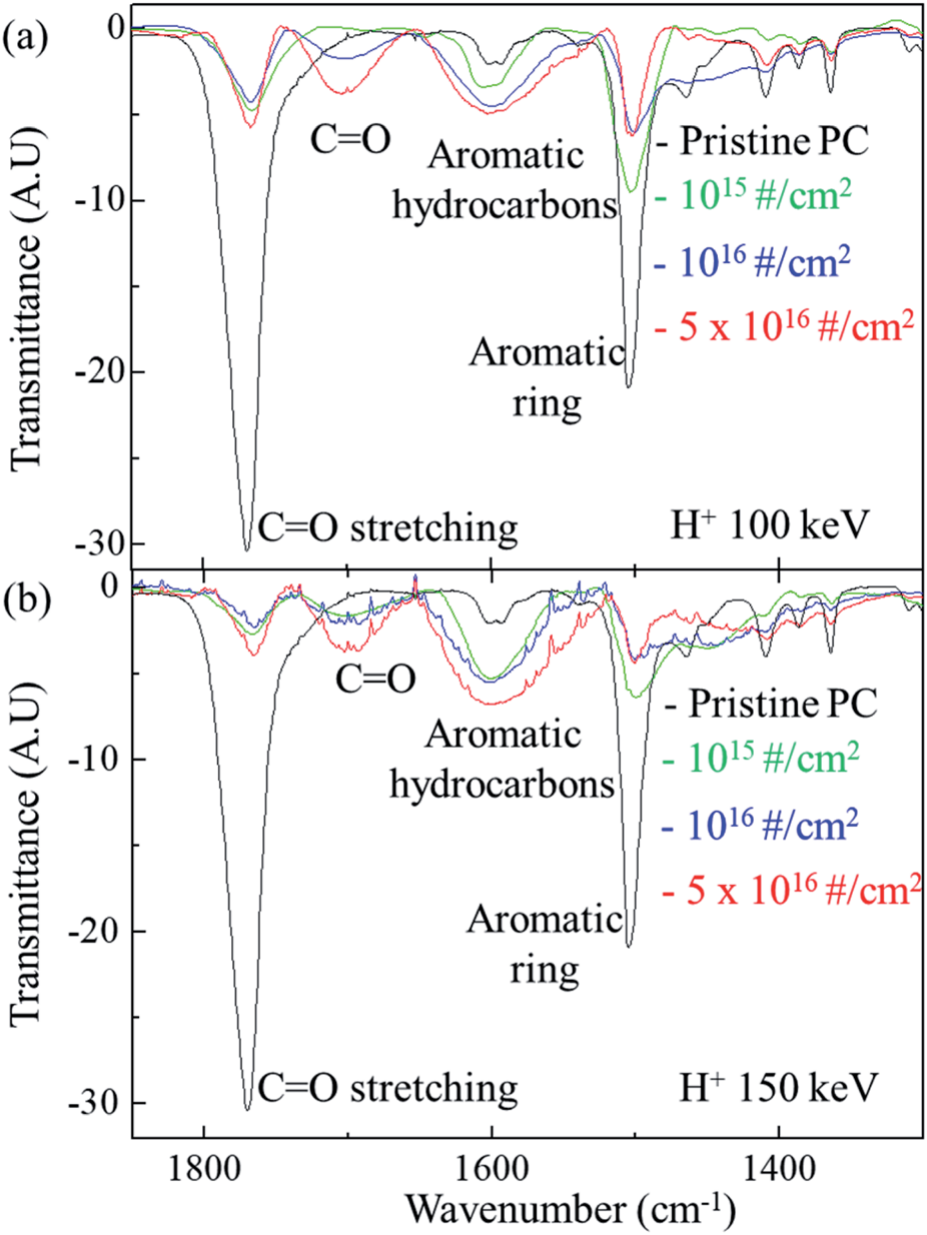
FTIR spectra change by H^+^ ion dose at the energy of (a) 100 keV and (b) 150 keV.

Fig. 3 shows the FTIR spectra of the original PC and H^+^ irradiated PC for different energy. For the irradiation energy of 100 keV, as shown in Fig. 3(a), the black, green, blue line, and red lines represent the FTIR spectrum of pristine PC, H^+^ irradiated PC with the dose of 10^15^ # cm^−2^, 10^16^ # cm^−2^, and 5 × 10^16^ # cm^−2^, respectively. The FTIR spectrum of the pristine PC has strong transmittance peaks at 1500 cm^−1^ and 1770 cm^−1^, which originated from the aromatic ring and C=O stretching vibration, respectively. However, H^+^ irradiation systematically suppresses two peaks of pristine PC; the higher H^+^ dose makes the two peaks lower. Moreover, the higher H^+^ dose produces a new peak at ∼1700 cm^−1^ and develops a greater aromatic hydrocarbon peak at ∼1600 cm^−1^. The new peak at ∼1700 cm^−1^ can be identified as another C=O stretching vibration.^16^ In fact, the carbon in the C=O bonds of pristine PC also forms bonds with other oxygen: C–O single bonds. The FTIR transmittance peak at ∼1770 cm^−1^ reflects all these connections. When H^+^ ions are irradiated, however, C–O single bonds are easily broken, compared with C=O double bonds, because the bonding energy of C–O is lower than that of C=O. Although bonds are broken by certain actions, they try to recover or form bonds with neighboring atoms; the higher H^+^ dose leads to a greater chance of C–H single bond formation. In other words, a new C=O stretching vibration peak for the H^+^ irradiated PC can be developed at a low wavenumber in the FTIR spectra. Indeed, H^+^ irradiation on PC develops a new C=O stretching vibration around 1700 cm^−1^ and suppresses C=O stretching vibration around 1770 cm^−1^, as shown in Fig. 3(a). This mechanism also explains the FTIR spectra for the PC irradiated by 150 keV (shown in Fig. 3(b)) and 200 keV (not shown here). In Fig. 3(b), the black, green, blue, and red lines represent the FTIR spectra of pristine PC, and H^+^ irradiated PC with the dose of 10^15^ # cm^−2^, 10^16^ # cm^−2^, and 5 × 10^16^ # cm^−2^, respectively.

In addition, H^+^ irradiation can also lead to an aromatic hydrocarbon peak around 1600 cm^−1^ in the FTIR spectra. Since aromatic hydrocarbons have sigma bonds, the bonding energy of an aromatic hydrocarbon is larger than that of an aromatic ring. Therefore, the transmittance peaks of the aromatic hydrocarbons have higher wavenumbers than those of aromatic rings in the FTIR spectra. Again, H^+^ irradiation supplies more chances to form aromatic hydrocarbons by rearranging the bonds of aromatic rings. Fig. 3(a) shows that the higher H^+^ irradiation dose causes the higher peaks for aromatic hydrocarbons and the lower peaks for aromatic rings at the energy of 100 keV. The same mechanism can be also applied to the FTIR spectra for the other energies such as 150 keV (shown in Fig. 3(b)) and 200 keV (not shown here).

**Fig. 4:**
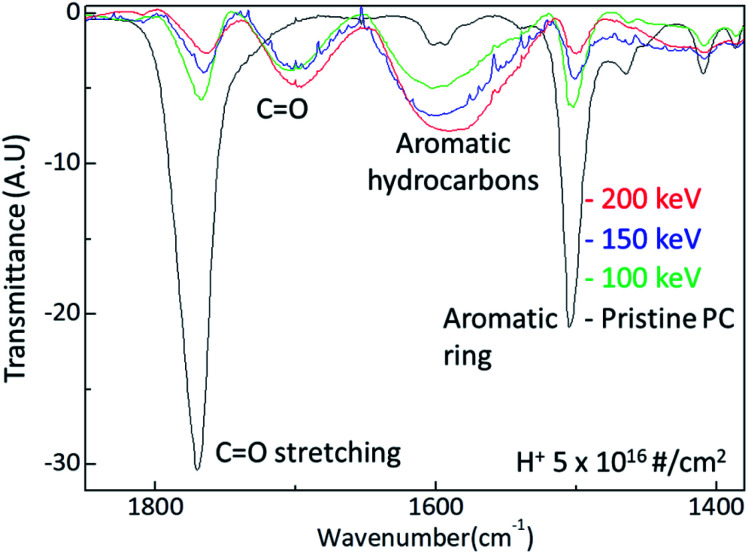
FTIR spectra change by H^+^ ion irradiation energy.

The surface hardness of H^+^ irradiated PC has energy dependence where the surface hardness increases with increasing irradiation energy. In order to certify the energy dependence, FTIR spectra were obtained from the H^+^ irradiated PC with different energy at the dose of 5 × 10^16^ # cm^−2^, as shown in Fig. 4. The black, green, blue, and red lines represent the FTIR spectra of pristine PC, and H^+^ irradiated PC with the energies of 100 keV, 150 keV, and 200 keV, respectively. Interestingly, by increasing the H^+^ irradiation energy, the transmittance peaks for C=O stretching vibration around 1770 cm^−1^ and aromatic ring decrease while the transmittance peaks for C=O stretching vibration around 1700 cm^−1^ and aromatic hydrocarbons are developed. In conjunction with the surface hardness shown in Fig. 1, the FTIR spectra change very systematically with increasing H^+^ irradiation dose and energy, as shown in Fig. 3 and 4. This means that surface hardness enhancement is related to the development of the new C=O stretching vibration around 1700 cm^−1^ and aromatic hydrocarbons formation. In other words, H^+^ irradiation produces a new C=O stretching vibration around 1700 cm^−1^ and forms more aromatic hydrocarbons, which is a reason for the hardness enhancement of H^+^ irradiated PC.

**Fig. 5:**
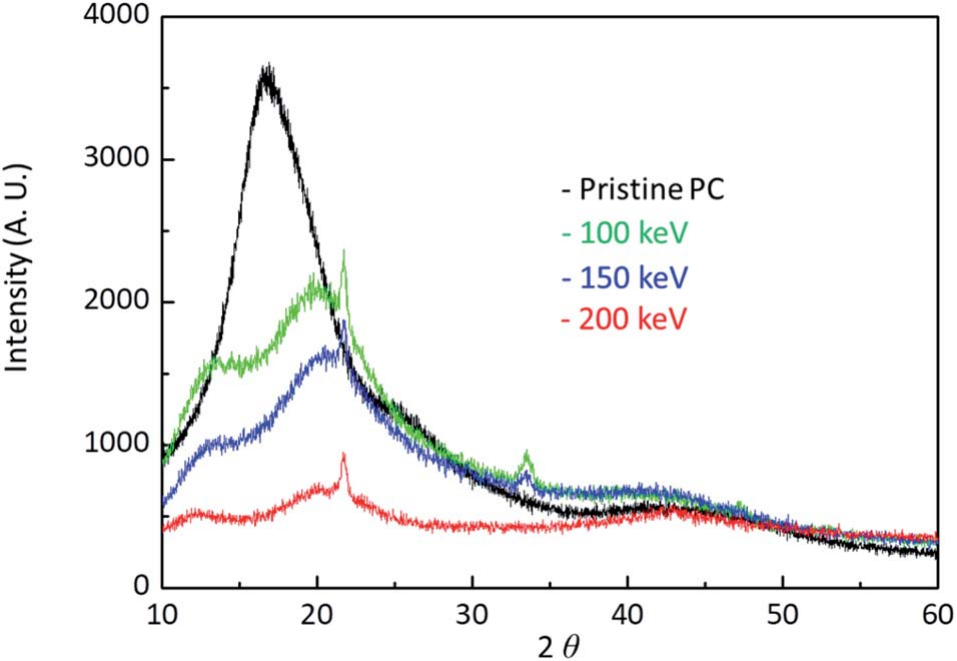
X-ray diffraction data for pristine PC and H^+^ irradiated PC with the energy of 100 keV, 150 keV, and 200 keV.

Fig. 5 shows the X-ray data of pristine PC and H^+^ irradiated PC. The black, the green, the blue, and the red lines are the X-ray data of pristine PC and H^+^ irradiated PC with the energies of 100 keV, 150 keV, and 200 keV, respectively. In order to compare energy dependency, the X-ray data were obtained from the same dose of 10^16^ # cm^−2^. It is known that X-ray diffraction for amorphous PC has a broad peak at 2*θ* = ∼17°,^17^ which is the same as pristine PC. Interestingly, the X-ray data for H^+^ irradiated PC show two peaks at low angles. This indicates that H^+^ irradiation changes the properties of PC through crosslinking and forming new C=O stretch bonds and aromatic hydrocarbons. Moreover, the broad peak intensity decrease with increasing the H^+^ irradiation energy, suggesting that H^+^ irradiation leads to the amorphous behaviour of pristine PC to become weak. Generally speaking, an amorphous state is softer than a crystal state, and thus, the X-ray behaviour is consistent with the surface hardness and FTIR spectra.

When energetic particles with the energy of 100 keV pass through polymers, they can break all the neighboring bonds whose energy are at most 10 eV. The broken bonds in a polymer chain can form bonds in other polymer chains, meaning that crosslinks occur by ion beam irradiation. In fact, it is known that crosslinks are a general reason to increase surface hardness. In this experiment, H^+^ irradiation also produces crosslinks between polymer chains in PC. In addition to crosslinks, H^+^ irradiation also generates new C=O stretch bonds and aromatic hydrocarbons. Consequently, the surface hardness enhancement by H^+^ irradiation results from all these effects.

## Conclusions

The surface hardness for H^+^ irradiated polycarbonate (PC) is examined by nano-indentation, FTIR, and X-ray. The nano-indentation test reveals that the surface hardness increases with increasing H^+^ irradiation dose and energy. The FTIR spectra are consistent with the nano indentation test. The higher dose develops a new C=O stretching vibration around 1700 cm^−1^ and aromatic hydrocarbons and thus hardened the surface of the H^+^ irradiated PC. In addition, the higher irradiation energy also develops a new C=O stretching vibration around 1700 cm^−1^ and aromatic hydrocarbons. Moreover, X-ray diffraction data are also consistent with surface hardness enhancement by H^+^ irradiation energy. Based on the experiments, the mechanism of the surface hardness enhancement for H^+^ irradiation PC is closely related to the formation of C=O stretching vibration around 1700 cm^−1^ and aromatic hydrocarbons.

## Conflicts of interest

There are no conflicts to declare.

## Supplementary Material
